# Flexible CNT-Interpenetrating Hierarchically Porous Sulfurized Polyacrylonitrile (CIHP-SPAN) Electrodes for High-Rate Lithium-Sulfur (Li-S) Batteries

**DOI:** 10.3390/nano14131155

**Published:** 2024-07-06

**Authors:** Jiashuo Shao, Cheng Huang, Qi Zhu, Nan Sun, Junning Zhang, Rihui Wang, Yunxiang Chen, Zongtao Zhang

**Affiliations:** School of Materials Science and Engineering, Zhengzhou University, Kexue Ave. 100, Zhengzhou 450001, China; zzusjs2021@163.com (J.S.); hc_1827465@163.com (C.H.); 18143973357@163.com (Q.Z.); sunnan20010315@163.com (N.S.); 13944291225@163.com (J.Z.); wangrihui23@163.com (R.W.); yxchen@zzu.edu.cn (Y.C.)

**Keywords:** lithium-sulfur batteries, sulfurized polyacrylonitrile (SPAN), phase separation, self-supporting, rate performance

## Abstract

Sulfurized polyacrylonitrile (SPAN) is a promising cathode material for lithium-sulfur batteries owing to its reversible solid–solid conversion for high-energy-density batteries. However, the sluggish reaction kinetics of SPAN cathodes significantly limit their output capacity, especially at high cycling rates. Herein, a CNT-interpenetrating hierarchically porous SPAN electrode is developed by a simple phase-separation method. Flexible self-supporting SPAN cathodes with fast electron/ion pathways are synthesized without additional binders, and exceptional high-rate cycling performances are obtained even with substantial sulfur loading. For batteries assembled with this special cathode, an impressive initial discharge capacity of 1090 mAh g^−1^ and a retained capacity of 800 mAh g^−1^ are obtained after 1000 cycles at 1 C with a sulfur loading of 1.5 mg cm^−2^. Furthermore, by incorporating V_2_O_5_ anchored carbon fiber as an interlayer with adsorption and catalysis function, a high initial capacity of 614.8 mAh g^−1^ and a notable sustained capacity of 500 mAh g^−1^ after 500 cycles at 5 C are achieved, with an ultralow decay rate of 0.037% per cycle with a sulfur loading of 1.5 mg cm^−2^. The feasible construction of flexible SPAN electrodes with enhanced cycling performance enlists the current processing as a promising strategy for novel high-rate lithium-sulfur batteries and other emerging battery electrodes.

## 1. Introduction

The dual-carbon goals have raised continuous worldwide demands for high-density energy storage systems [1]. Nowadays, batteries are irreplaceable in many practical fields, such as automobiles, portable electronics, and aerospace [2]. As one of the most promising battery systems, lithium-sulfur (Li-S) batteries have been developed and widely investigated as early as the 1960s due to their high energy density, low cost, and resource abundance [3]. The theoretical specific energy density for Li-S can reach up to 2600 Wh kg^−1^, almost five times higher than that of commercial Li-ion batteries [4,5]. However, issues such as the shuttle effect of long-chained polysulfides (PSs), limited high-rate cycling stability, lithium dendrite formation, and sulfur cathode volume expansion should be addressed before practical applications [6,7,8]. Sulfurized polyacrylonitrile (SPAN), an important candidate in Li-S battery systems with representative short-chained solid-to-solid conversion and lower self-discharge, is regarded as one potential choice to solve the problems for Li-S batteries [8,9,10]. In recent years, there has been a growing trend in the investigation of lithium SPAN battery systems in research and commercial areas.

The molecular structure of SPAN is characterized by the cyclization of the –CN group into a more stable conjugated polypyridine ring containing C=C and C=N double bonds, where the sulfur atoms are attached to the main chain by covalent bonds such as C–S and S–S. Unlike the conventional Li-S battery system with long-chained polysulfides (Li_2_S_n_, 8 ≥ n ≥ 4), the electrochemical reaction of lithium and SPAN results in a solid–solid conversion between the short-chained Li_2_S_2–4_ [11,12], and only one charge–discharge plateau at around 1.9 V can be observed during the electrochemical cycling [13,14]. Also, SPAN can show better oxidation resistance and stability for higher safety compared with S [15]. However, SPAN is usually recognized as a good ionic conductor (conductivity of 10^−4^ to 10^−3^ S cm^−1^) but a poor electron conductor, which lags the total redox reaction kinetics. Also, the content of sulfur is highly limited by the cyclization reaction of SPAN, which results in relatively low sulfur content (i.e., ~45% for theoretical limitation and ~30% among existing reports [16]. Systematic optimizations of the electrode compositions, ion/electron transportation channels, and electrode/electrolyte interfaces are urgently required to prompt practical applications of SPAN-based batteries [17].

Many strategies have been developed to address these issues, including building porous electrodes, low-dimensional composites, introducing catalysts, etc. [18,19,20,21]. Among these methods, the building of porous structures is quite advantageous, which can not only enhance the accessibility of electrolytes but also significantly facilitate the ion transfers between the electrode and electrolyte. Additionally, the incorporation of large amounts of pore structures can optimize sulfur utilization and increase the specific capacity. Furthermore, the microporous structure can avoid direct contact between the electrolyte solvent and the internal sulfur, which can inhibit the formation and dissolution of polysulfides, thereby suppressing sulfur loss during continuous cycling processes [22,23]. Liu et al. [20] propose an electrospinning-based method for the fabrication of self-supporting porous SPAN composite cathodes, which show a high Coulombic efficiency and an excellent specific capacity as high as 903 mAh g^−1^ after 150 cycles at 1 C. However, the process also faces challenges of high cost and a cumbersome process for production. Moreover, the electrochemical reaction kinetics in SPAN cells is limited due to the inherent properties of the SPAN molecule. To address this, researchers have proposed the incorporation of a catalytic mechanism, which can effectively reduce the reaction activation energy and accelerate the conversions by catalytic compositions, such as MoS_2_ [21], CoSe_2_ [24], and CoS_2_ [25]. However, with long-term cycling of the batteries, the introduction of catalytic metal compounds can not only cause side reactions such as the polarization effect, metal ion dissolution, and polysulfide precipitation, thus affecting the cycling stability, but also elevate the costs of large-scale production. Moreover, there are few reports focusing on the feasible synthesis of flexible electrodes that can contribute to better mechanical stability and adaptability, which are particularly important for applications in wearable and flexible electronic devices. Therefore, the rational design and integration of pore structures in SPAN electrodes, aiming to significantly enhance their electrochemical properties, remains quite challenging.

In this work, a novel self-supporting CNT-interpenetrating hierarchically porous sulfurized polyacrylonitrile (CIHP-SPAN) composite electrode is developed by a facial cost-effective phase separation method. Flexible SPAN electrodes with excellent electron and ion conductivity can be synthesized directly, eliminating the need for binders or additional complex treatments. The obtained electrodes show a hierarchical pore size distribution (micro-, meso-, and macro-pores), which optimizes sulfur loading and enhances energy storage characteristics [26]. Also, the direct growth of SPAN on interconnected CNT drastically boosts the electrical connections, mitigating the sluggish reactions [27]. Impressively, at a sulfur loading of 1.5 mg cm^−2^, an initial specific capacity of 1090 mAh g^−1^ and a retained capacity as high as 800 mAh g^−1^ can be obtained at 1 C for 1000 cycles. To further elevate the high current density cycling performance of the CNT-interpenetrating hierarchically porous sulfurized polyacrylonitrile electrode, a V_2_O_5_/carbon fiber (V-CF) interlayer is introduced, where a high initial capacity of 1003.3 mAh g^−1^ and a remarkable sustained capacity of 860 mAh g^−1^ after 610 cycles at 2 C can be obtained, demonstrating a low-capacity loss rate of only 0.022% per cycle. Also, a high initial capacity of 614.8 mAh g^−1^ and a notable sustained capacity of 500 mAh g^−1^ after 500 cycles at 5 C, with a single-cycle loss rate of 0.037% can be obtained. The integrations of a hierarchically porous structure and electron/ion conductive pathways in binder-free electrodes by the simple phase separation and annealing method, as reported in this work, can open up new perspectives for building a variety of high-performance electrodes for Li-S and other emerging batteries.

## 2. Materials and Methods

### 2.1. Chemicals and Materials

All chemicals were commercially available and used as received. Polyacrylonitrile (PAN) (Mw = 150,000) and polyvinyl pyrrolidone (PVP) were purchased from Sigma-Aldrich Co., Ltd., Shanghai, China. Sulfur (S, ≥99.5 wt%) and ethyl alcohol were provided by Shanghai Chemical Corporation (Shanghai, China). N, N-Dimethylformamide (DMF) was purchased from Sinopharm Chemical Reagent Co., Ltd., Shanghai, China. N-Methy-2-pyrrolidone (NMP, 99.99 wt.%), polyvinylidene fluoride (PVDF, ≥99.5 wt.%), 1,3-dioxolane (DOL), 1,2-dimethoxyethane (DME) and lithium bis(trifluoromethanesulfonyl) imide (LiTFSI) were bought from Alfa Aesar (Haverhill, MA, USA). Multi-walled CNT was bought from the Chinese Academy of Sciences, Chengdu Organic Chemistry Co., Ltd., Chengdu, China. All chemicals and solvents are analytical grade.

### 2.2. Preparation of SPAN and CNT Interpenetrating SPAN Electrode

The SPAN composite was synthesized by directly pyrolyzing PAN and sulfur composite powders. Typically, PAN (Mw = 150,000) and sulfur powders are mechanically mixed in a mortar at room temperature in a mass ratio 1:4 for 2 h. After that, the mixture is sealed in a small vacuum quartz tube in Ar atmosphere and heated at 350 °C for 6 h [28].

For the CNT-interpenetrating SPAN electrode, S and PAN mixture (S:PAN = 4:1), PVP and CNT were stirred in DMF solution by magnetic stirring for 6 h at room temperature, and coated on a coating machine at a thickness of 200–400 μm to form a precursor membrane. After that, the as-obtained membranes were immediately immersed in deionized water to trigger the phase separation process and further ultrasonicated for about 10 min to form hierarchical pores. The obtained mixture membranes were then sealed in a vacuum quartz tube furnace and heated up 350 °C for 6 h to obtain the CNT-interpenetrating SPAN electrode. The CNT and SPAN composite samples are noted as CIHP-SPAN, with a CNT loading amount of 10%. Samples without PVP addition during CIHP-SPAN sample preparation are noted as CNT-SPAN.

### 2.3. Synthesis of V_2_O_5_/Carbon Fiber (V-CF) Interlayers

To enhance the high rate cycling stability of the cell, we developed a novel hydrothermal method for the synthesis of V_2_O_5_/carbon fiber interlayers [26]. This method involved the synthesis of carbon fibers (CFs) by an electrostatic spinning of the PAN in DMF solution onto aluminum foil, which was further annealed in an air atmosphere at 280 °C for pre-oxidization of the PAN, followed by carbonization at 900 °C in N_2_. Subsequently, a hydrothermal method was applied to deposit vertically aligned V_2_O_5_ nanoflakes onto the CFs. Detailed deposition parameters can be found in the Supporting Information.

### 2.4. In Situ UV–Vis Absorption Spectra

An in-situ UV–vis spectrometer was assembled by fixing the CNT-interpenetrating hierarchically porous sulfurized polyacrylonitrile cathode and lithium foil anode to two sides of a cuvette with conductive adhesive tape. The separators without and with V-CF interlayers were covered on the cathode side. The entire assembly process was carried out under an argon atmosphere, and the cell was sealed before the measurements were conducted.

### 2.5. Characterizations

The morphologies of the samples were characterized by scanning electron microscopy (SEM, Auriga FIB, Carl Zeiss, Oberkochen, Germany) in secondary electron imaging (SEI) mode. The compositions of different samples were characterized by X-ray photoelectron spectroscopy (XPS) spectra performed on a PHI Quantera SXM (ULVAC-PHI, Chigasaki, Kanagawa, Japan) system with an Al/K anode (photon energy = 1486.6 eV) mono X-ray source. A powder X-ray diffraction (XRD) pattern was collected on an X-ray diffractometer (DX-2700BH, Dandong Haoyuan, Dandong, Liaoning, China) using Cu Kα as the radiation source. Raman spectroscopy was measured by a Confotec MR520 instrument (Graben, Graben-Neudorf, Germany) with an excitation wavelength of 532 nm. Thermogravimetric (TG) analysis was performed under nitrogen flow using a thermal analyzer apparatus (STA449F3, NETZSCH, Selb, Germany) with a heating rate of 10 °C/min from room temperature to 1000 °C. Nitrogen adsorption–desorption isotherms were recorded on an ASAP 2460 (Micromeritics, Norcross, GA, USA) apparatus at a temperature of 77 K. The specific surface area and the pore structure were measured by the nitrogen sorption using a physisorption analyzer (JW-BK112, Beijing JWGB, Beijing, China). The optical absorption of the samples was obtained by using a UV-1600PC (Shanghai Aoyi, Shanghai, China) instrument in the wavelength of 200–780 nm. The content of C, H, O, N, S and other elements in SPAN was detected with an organic elemental analysis test (EA) (Leeman EA3000, Elementar, Langenselbold, Germany).

Electrochemical measurements: The as-prepared CIHP-SPAN cathodes were assembled as CR2032 coin cells with a lithium anode and electrolyte. The electrolyte was made of 1 M lithium bis(trifluoromethanesulfonyl)imide (LiTFSI) and 0.1 M LiNO_3_ dissolved in 1,3-dioxolane (DOL) and 1,2-dimethoxyethane (DME) solution (1:1 *v*/*v*). Celgard2325 (Celgard LLC., Charlotte, NC, USA) or V-CF interlayer coated Celgard2325 were used as the separator for corresponding cells. Galvanostatic charge/discharge (GCD) and rate studies were performed on a battery test system (LANDCT2001A, Landdian, Wuhan, China) with voltages ranging from 1 to 3 V (vs. Li/Li^+^) at different cycle rates. Cyclic voltammetry (CV) was carried out on an electrochemical workstation (PGSTAT302N, Metrohm, Herisau, Switzerland) with scan rates of 0.1–0.5 mV s^−1^. Specific charge/discharge capacities were calculated based on the mass of sulfur in the SPAN. The working electrode’s electrochemical impedance spectroscopy (EIS) was recorded on an electrochemical workstation in the frequency range of 10^−2^–10^5^ Hz.

## 3. Results

**Figure 1:**
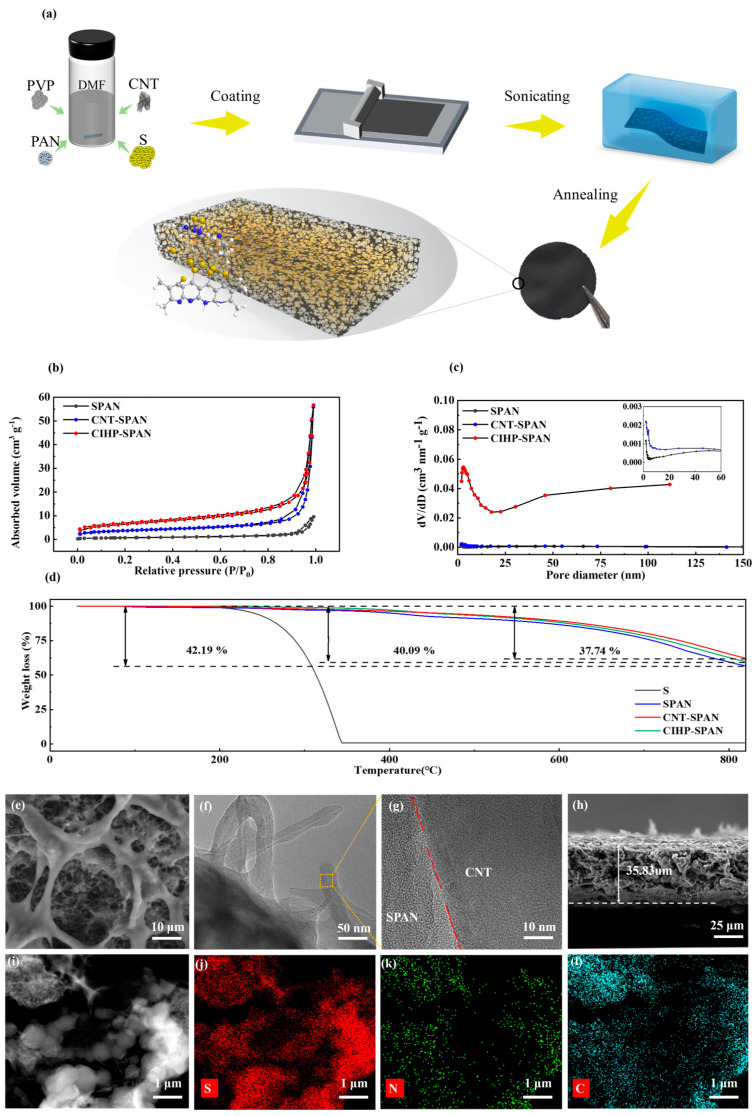
(**a**) Schematic diagram for the synthesis of CIHP-SPAN cathode materials. In the molecular structure diagram (the bottom figure of (**a**)), the yellow, blue, gray, and white spheres represent sulfur, nitrogen, carbon, and hydrogen atoms, respectively. (**b**) N_2_ adsorption/desorption isotherms and (**c**) pore size distributions of SPAN, CNT-SPAN and CIHP-SPAN composites. (**d**) Thermogravimetric analysis (TGA) of S, SPAN, and CIHP-SPAN composites. SEM (**e**) and TEM (**f**) image of the CIHP-SPAN sample. (**g**) High-resolution TEM image for CIHP-SPAN sample taken at the selected position of figure (**f**). Cross-sectional comparison of CIHP-SPAN anode composite (**h**) after cycling for 200 revolutions. (**i**) STEM images of CIHP-SPAN composites and (**j**–**l**) the energy dispersive X-ray spectroscopy (EDS) elemental mapping of S, N, C.

Phase separation is an effective method for the synthesis of hierarchical pore structures. As shown in Figure 1a, a precursor casting solution was obtained by blending the mixtures of CNT, S, PVP, and PAN in DMF solvent through vigorous stirring. As both PVP and PAN are highly soluble in DMF solution, they can work as the film-forming agent to facilitate the coating of uniform precursor films. However, upon immersing the precursor films in a water bath, drastic phase separation and aggregation of the PAN components (mixed with S and CNT) occurred due to the low solubility of PAN polymer in water [26]. PVP can be subsequently removed by water during ultra-sonicating, forming large amounts of micro-, meso- and nano-scale hierarchical pores in the final products. PAN can be chemically bonded to sulfur to form the SPAN after annealing at 350 °C in N_2_. It is noteworthy that CNT and S can be evenly distributed in the solution by ultra-sonicating and form homogenous distributions in the SPAN materials after annealing. Hierarchically porous SPAN electrodes with interpenetrated CNT conduction networks can be formed. As shown in Figure 1b, both the phase separation process and the addition of CNT lead to an increase in the adsorption/desorption of nitrogen from the samples. Significantly increased amounts of micropores (<2 nm), mesopores (2~50 nm), and large nano-/macro-pores (>50 nm) can be found for samples with PVP addition (Figure 1c). These multiscale pore structures are essentially beneficial for improving electrode performance. This is because the micropores (<2 nm) can provide a large surface area with reactive sites, but the infiltration of electrolyte ions into these pores is rather difficult causing low kinetics; the transportation of ionic species in meso- (2–50 nm) and macro- (>50 nm) pores is much easier, but their surface-to-volume ratios are not high enough. A hierarchical porous structure with broadened pore-size distribution is preferred to enhance the electrochemical properties. The calculated specific surfaces of the SPAN and CNT-incorporated SPAN (denoted as CNT-SPAN) composite samples are 12.8902 and 24.6125 m^2^ g^−1^, respectively, which is drastically increased to 50.4319 m^2^ g^−1^ for the CNT-interpenetrating hierarchically porous SPAN (denoted as CIHP-SPAN) with PVP addition (see Table S1 in Supporting Information). The addition of PVP increases the samples’ specific surface area and improves the multiscale pores’ content, which can enhance the electrochemical properties, as discussed in the following sections.

Thermogravimetric analysis (TGA) is carried out to investigate the influence of phase separation on the as-obtained electrode materials, as shown in Figure 1d. It can be seen that the weight loss of S ends at about 200 °C, following the typical TGA curve as reported. For SPAN, CNT-SPAN, and CIHP-SPAN, the sulfur content is about 42.19%, 40.09%, and 37.74%, respectively, similar to results from the organic elemental analysis (EA) in Table S1 [29]. This indicates that the hierarchical porous structure has a more substantial binding capacity for sulfur. TEM and high-resolution TEM (HRTEM) images (Figure 1f,g) provide further evidence that amorphous SPAN is closely deposited on the CNT surfaces, thereby ensuring excellent electrical contact for electrochemical cycling. Cross-section SEM characterization for the CIHP-SPAN electrode after 200 cycles (Figure 1h) indicates an intact carbon skeleton structure after cycling. Energy dispersive X-ray spectroscopy (EDS) analysis (Figure 1j–l) further shows that detected signals of S, C and N are uniformly distributed in the composites. The hierarchically porous structure of SPAN with interconnected carbon nanotube networks can enhance sulfur utilization and optimize the electron and ion conductivity pathways to achieve high electrochemical properties, which will be discussed in the subsequent sections. Moreover, the mechanical test in Figure S1 (Supporting Information) indicates that the composite electrodes are flexible and durable for large-angle bending, which is critically important in the fields of wearable and flexible devices.

**Figure 2:**
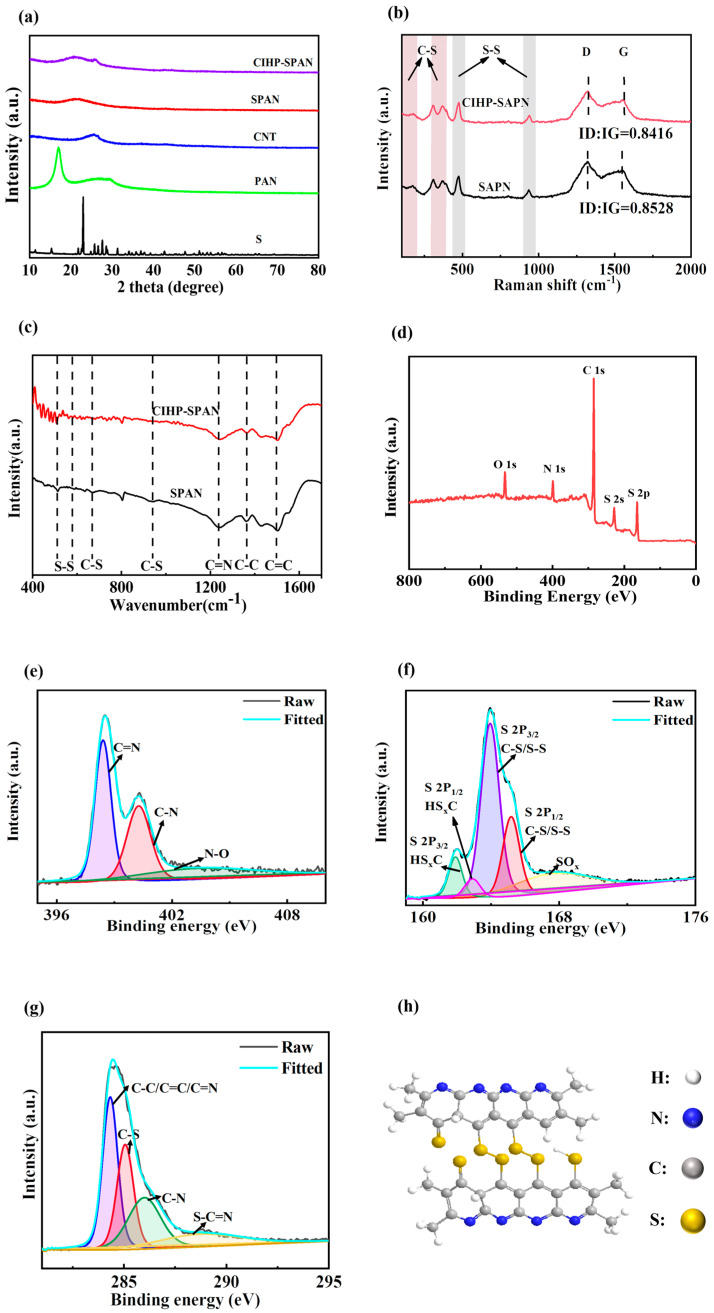
(**a**) X-ray diffraction (XRD) patterns for S, PAN, CNT, SPAN, and CIHP-SPAN composites. Raman (**b**) and infrared (**c**) spectra of SPAN and CIHP-SPAN composites, (**d**) X-ray photoelectron spectroscopy (XPS) survey spectra for the CIHP-SPAN composites, and high-resolution XPS spectra for S 2p (**e**), N 1 s (**f**) and C 1 s (**g**). (**h**) The molecular structure of SPAN.

The X-ray diffraction (XRD) patterns for different samples are shown in Figure 2a. Clear diffraction peaks assigned to sulfur, PAN and CNT can be found for pure samples of S, PAN and CNT. For the SPAN and CIHP-SPAN samples, the broad diffraction peak at·2θ = 20°~30° could be ascribed to the overlapping of the graphene characteristic peak (002) and the amorphous SPAN peak. Characteristic diffraction peak for CNT (2θ ≈ 26°) can also be found in the CIHP-SPAN, indicating the formation of SPAN and CNT composites in the sample. Raman spectra of the SPAN and CIHP–SPAN samples are shown in Figure 2b. Characteristic Raman modes of C–S bonds are located at around 176, 308, and 370 cm^−1^, and the characteristic peaks of S–S bonds are located at around 464 and 932 cm^−1^, respectively. An ambiguous peak at 1458 cm^−1^ comes from C–C and C–S stretching [30,31,32,33]. All the tested samples have D and G peaks corresponding to the carbon structure. Specifically, the D peak is mainly located at 1343 cm^−1^, which reflects the degree of carbon defects and crystalline disorder; the G peak is mainly located at 1606 cm^−1^, which is mainly caused by the in-plane vibration of sp2 hybridized carbon atoms, and the existence of the G peak indicates that the synthesized carbon materials are partially graphitized. Furthermore, the ratio of ID/IG reflects the degree of graphitized carbon material. Combined with the Raman data, it can be seen that the ID/IG values of SPAN and CIHP-SPAN samples are 0.8528 and 0.8416, respectively, indicating that the CIHP-SPAN samples have a better graphitization.

**Figure 3:**
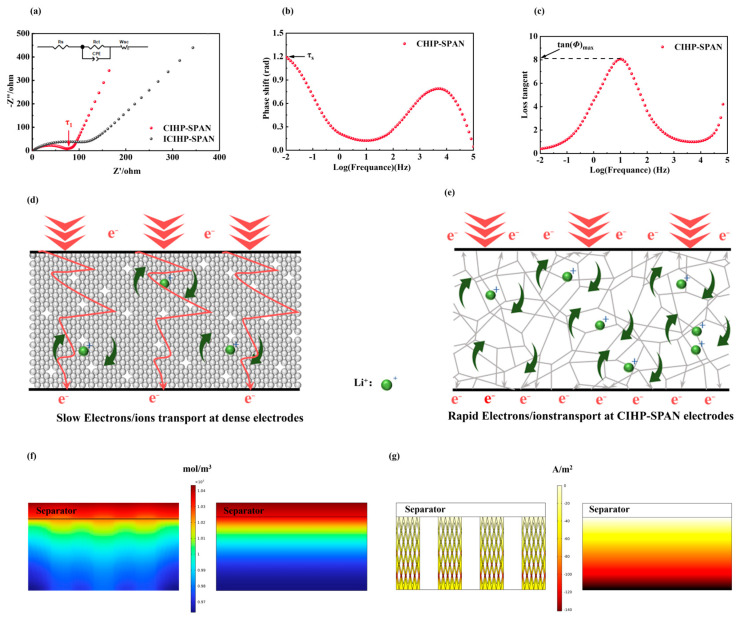
(**a**) EIS and simulated Nyquist charts for SPAN and CIHP-SPAN electrodes. (**b**) Bode plot of the CIHP-SPAN electrode. (**c**) Loss tangent plot of the CIHP-SPAN electrode. Schematic diagrams of electron–ion conductance analysis of a conventional SPAN electrode (**d**) and CIHP-SPAN electrode (**e**). (**f**) Simulated electrolyte concentration distribution at the discharge end for CIHP-SPAN (left) and SPAN electrodes (right) at 50 A/m^2^ current density. (**g**) Interfacial reaction current density distribution at the end of discharge at 50 A/m^2^ current density for CIHP-SPAN (left) and SPAN electrodes (right), respectively.

Fourier transform infrared spectroscopy (FTIR) was also performed to investigate the structure of the CIHP-SPAN composites further and understand the chemical structure of the samples (in Figure 2c). As shown in the FTIR results, the absorption bands that appeared at 943 and 635 cm^−1^ can be attributed to the C–S bonds, while the absorption at 513 cm^−1^ can be assigned to the S–S vibration. X-ray photoelectron spectroscopy (XPS) in Figure 2d further shows four elemental signals belonging to the S, C, N and O, respectively. The high-resolution C1s XPS spectrum (Figure 3e) can be divided into four peaks centering at 284.7, 285.6, 286.7, and 288.7 eV, which can be assigned to C–C/C=C/C=N, C–S, C–N, and C=O bonds, respectively [34,35]. As shown in Figure 2f, the S2p spectrum can be fitted into five components. Specifically, the peaks centered at 163.5 eV (S 2p3/2) and 164.8 eV (S 2p1/2) are associated with the vibrations for C–S and S–S bonds, respectively. The other two XPS peaks centered at 161.7 eV (2p3/2) and 163.0 eV (S 2p1/2) can be attributed to the adsorption signals from the by-product HS_x_C formed in the sulfidation reaction. The peak observed at 167.6 eV is also assigned to the oxidized S species (e.g., SO_x_). Regarding the N signal (Figure 2g), the two XPS peaks centered at 398.3 eV and 400.1 eV can be assigned to the C=N and C–N bindings in the molecular of PAN, whereas an additional weaker peak at 403.1 eV can be indexed to the presence of N–O bonds. Combined with Raman and XPS results, it is inferred that covalent S exists in CIHP-SPAN complexes in the form of C–S and S–S bonds, corresponding to the proposed molecular structure, as shown in Figure 2h [23,36].

Asymmetrical cells are assembled using lithium foil as the anode and SPAN composite electrodes as the cathodes to investigate the electrochemical properties. Electrochemical impedance spectroscopy (EIS) in Figure 3a shows the typical spectrum profile with two characteristic parts, i.e., a semicircle at the high-frequency region and a slash at the low-frequency region, which are attributed to the charge transfer resistance (*R*_ct_) and Warburg impedance of the cells, respectively. The obtained *R*_ct_ for SPAN and CIHP-SPAN are 215.8 Ω and 66.45 Ω, respectively (Table S2) [37,38,39]. Due to the excellent contact between CNT and SPAN, as shown in the TEM images (Figure 1f,g), the *R*_ct_ of the cell is suppressed significantly. In addition, the slope in the low-frequency region for the CIHP-SPAN sample is much higher than that of the SPAN, showing an improved ionic conductivity due to hierarchical pores and the large specific surface area that are beneficial for ion penetration and transportation. Moreover, as shown in the EIS spectra, the cells with SPAN and CIHP-SPAN cathodes show intercepts of 4.4 Ω and 3.4 Ω, respectively, on the real axis (*Z*′), which corresponds to the composite resistance (*R*_e_).

In order to study the kinetics of CIHP-SPAN electrodes, the Li^+^ diffusion coefficients (*D_Li_*) are calculated according to the impedance spectroscopy data [40]. The *D_Li_* of the CIHP-SPAN electrode can be calculated by the diffusion length (*l*_D_) and the diffusion time constant (*τ*_D_), according to Equation:

*D_Li_* = *l*_D_^2^/*τ*_D_

The diffusion length is based on the following equations:

*l*_D_ = √*τ_S_* × √*D*_m_

*D*_m_ = *λ*_D_^2^/*τ*_1_

*λ*_D_ = *d*/*δ*

tan(*Φ*)_max_ = √*δ/2*

The *τ*_D_ can be obtained from *W_SC_* following the equation:

*τ*_D_ = *W*_SC_^2^

in which *D*_m_ and *λ*_D_ are the diffusion coefficient of net mobility and the Debye length of electrode, respectively; *δ* is a dimensionless number; *τ*_1_ is the reciprocal of the frequency corresponding to the inflection point of the Nyquist plot; *τ_S_* is the reciprocal of the frequency corresponding to the highest point below 0.01 Hz in Bode plot; tan(*Φ*)_max_ can be obtained from loss tangent plot; *d* is the half thickness of the electrode; *W*_SC_ is a part of the Warburg O-element, which can be extracted from the simulated equivalent circuit diagram.

According to the equations above, the calculated *D*_Li_ of SPAN and CIHP-SPAN electrodes are 4.23 × 10^−11^ and 2.78 × 10^−10^, respectively. Notably, the significant enhancement in charge transfer resistance and *D*_Li_ indicates that the conductive hierarchically porous electrodes exhibit superior electronic and ionic conductivity, thereby ensuring improved rate performance. As shown in Figure 3d, the ion transport in conventional electrodes is hindered by the closely packed active materials and conductive carbon. In this configuration, Li^+^ ions are expected to traverse the electrodes quite slowly, bypassing the particles during transportation, whereas electrons are conducted efficiently within the dense conductive particles. In contrast, the CIHP-SPAN electrode poses a hierarchical porous structure that provides a direct, short-range and efficient pathway for Li^+^ ion conduction. Additionally, the interconnected three-dimensional conductive network ensures a continuous conductive channel for electron transport, further enhancing the electrode’s performance.

To investigate the influences of the hierarchical porous CNT-interpenetrating frameworks on the electrochemical performance of Li-S, electrochemical simulations of the CIHP-SPAN and conventional SPAN electrodes were performed by Multiphysics, the results of which are shown in Figure 3f,g. As shown in Figure 3f, the electrolyte inside the CIHP-SPAN electrode is more uniform at a stimulated current density of 50 A/m^2^. In contrast, the electrolyte concentration gradient of the conventional dense electrode is larger. As shown in Figure 3g, the hierarchical porous CIHP-SPAN electrode exhibits a more uniform current density distribution at a current density of 50 A/m^2^. In contrast, the conventional dense electrodes show a more considerable current density difference between the two sides [29,41]. These results indicate that the electrochemical reaction of the porous three-dimensional conductive network of the CIHP-SPAN electrode is much more homogeneous than the conventional dense electrode [42,43].

The cyclic voltammetry (CV) curves for SPAN and CIHP-SPAN are shown in Figure S3a,b (in Supporting Information). In the first cycle, a reduction peak is observed at 1.3 V because of the high activation energy required to break S–C or S–N covalent bonds [44,45]. The current density of the CIHP-SPAN electrode with a hierarchical porous CNT network is drastically enhanced in the two subsequent cycles. The current density of the SPAN electrode is minimal in comparison. The electrochemical properties were further investigated by varying the scan rates of the CV curves from 0.1 to 0.4 mV s^−1^, where a linearly related relationship to the square root of the scan rate is found, demonstrating a diffusion-controlled process [46,47,48].

**Figure 4:**
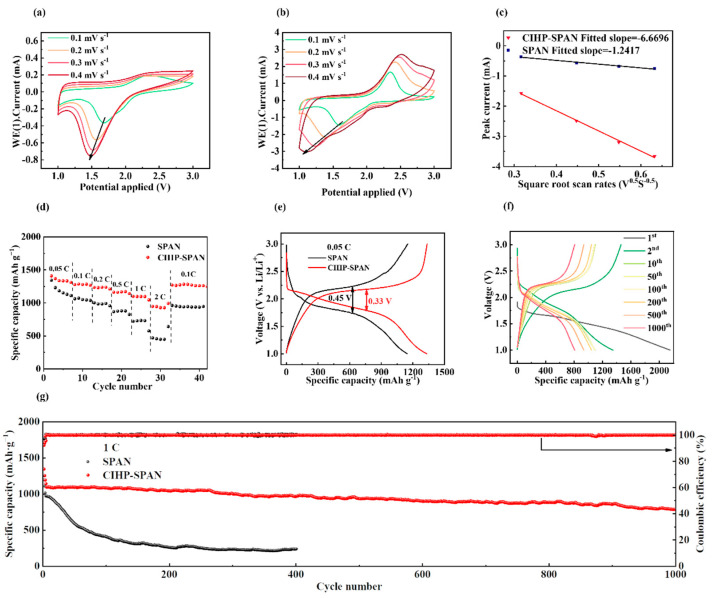
(**a**) Cyclic voltammetry (CV) plots of SPAN and (**b**) CIHP-SPAN at a scan rate of 0.1–0.4 mV s^−1^. (**c**) SPAN and CIHP-SPAN anodic reduction peak-to-peak currents versus scan rate. Rate properties (**d**) of SPAN and CIHP-SPAN composite cathode materials at specified current densities and their correlated charge and discharge curves at 0.05 C (**e**). (**f**) Voltage–capacity curves for a different number of cycles and (**g**) long cycling performance of SPAN and CIHP-SPAN at 1 C.

The diffusion behavior of Li^+^ ion can be evaluated by the Randles–Sevcik equation below:

*I*_p_ = 2.69 × 10^5^ n^1.5^ *A D*^0.5^
*C v*^0.5^ (25 °C)

where *I*_p_ represents the peak current intensity (mA), *n* is the number of electrons transferred (*n* = 2), *A* is the electrode area (cm^−2^), *D* represents the diffusion coefficient of Li^+^ (cm^2^ S^−1^), C represents the Li^+^ concentration (mol L^−1^), and *v* represents the scan rate (V s^−1^). The slope between *I*_p_ and *v*^0.5^ reflects the diffusion rate of Li^+^, which provides valuable information for the electrode reaction kinetics [47,49]. As shown in Figure 4c (the comparison of reduced peaks), the absolute value of the slope of CIHP-SPAN (*I*_p_/*v*^0.5^) is much larger than that of SPAN, which is consistent with the *D*_Li_ conclusions above, indicating that the structure of CIHP-SPAN is more favorable for the fast Li^+^ ion transport. This faster diffusion is mainly related to the formation of a hierarchical porous carbon nano skeleton, which provides more Li^+^ ion channels and facilitates the lithiation and delithiation process.

As shown in Figure 4d, the discharge capacities for CIHP-SPAN electrodes at currents of 0.05, 0.1, 0.2, 0.5, 1, and 2 C (1C = 1675 mA g^−1^) are 1333 mAh g^−1^, 1278.6 mAh g^−1^, 1231.3 mAh g^−1^, 1167.1 mAh g^−1^, 1097.7 mAh g^−1^, and 928 mAh g^−1^, respectively. In contrast, the SPAN-based electrode cells have rather low discharge capacity at both low and high current densities. Also, the CIHP-SPAN electrodes retain a high capacity after switching back to 0.1 C, showing excellent stability. Figure 4e shows the charge–discharge curves of the two composite electrodes of SPAN and CIHP-SPAN at a current of 0.05 C, with median voltage differences of 0.45 V and 0.33 V, respectively, showing significantly lowered polarization for samples with PVP-introduced hierarchically porous structures. The suppressed median voltage difference for the CIHP-SPAN electrode is essentially beneficial for the enhancement of electrochemical reactions. The long-cycle performances of the CIHP-SPAN at high current densities of 1C are shown in Figure 4f,g. The initial discharge capacity of the CIHP-SPAN electrode cycled at 1C is 1090 mAh g^−1^, while the discharge capacity remains 800 mAh g^−1^ after 1000 cycles, corresponding to a low-capacity loss of 0.026% for a single cycle. Coulombic efficiency is extremely erratic at a high current density of 5 C for both SPAN and CIHP-SPAN, as shown in Figure S4a (Supporting Information), which is attributed to the thermodynamic instability at the interface between Li-Metal and an organic electrolyte at a high current density, where the electrode potential of Li-Metal exceeds the range of the organic electrolyte, resulting in the reduction and decomposition of the electrolyte. Figure S4b,c show that the cycling performance of the SPAN sample exhibits remarkable cycling stability. Specifically, at 2 C, it achieves an initial capacity of 950 mAh g^−1^ and maintains a capacity of 720.8 mAh g^−1^ after 620 cycles, demonstrating a capacity decay rate of only 0.039% per cycle. Furthermore, at 0.5 C, the CIHP-SPAN starts with an initial specific capacity of 1090 mAh g^−1^ and, after 950 cycles, retains a capacity of 667.5 mAh g^−1^, corresponding to a capacity retention of 72.76%. These results indicate the superior cycling performance of the CIHP-SPAN electrodes.

To further investigate the electrochemical properties of CIHP-SPAN composites, we examined their cycling performance by varying sulfur loadings of 2.82, 5.46, 9.14, and 10.53 mg cm^−2^, respectively, while keeping the electrolyte quantity constant at 20 μL mg^−1^ of sulfur for comparison (Figure S4b, in Supporting Information). Notably, even with a high sulfur loading of 10.53 mg cm^−2^, the initial capacity remains impressive at approximately 675.1 mAh g^−1^. In addition, to gain a deeper understanding, we further test the cycling performance by adjusting the electrolyte content at a sulfur loading ranging from 1.4 to 1.6 mg cm^−2^ (Figure S4c). Specifically, electrolyte amounts of 15, 10, and 25 μL mg^−1^ of sulfur are applied. Remarkably, even with a reduced electrolyte content of 5 μL mg^−1^, the battery exhibits stable cycling for up to 200 cycles, although a significant decline in discharge capacity is observed after approximately 120 cycles. Battery disassembly experiment is performed for the CIHP-SPAN sample after 400 cycles at 2 C (Figure S5, in Supporting Information). We can see apparent corrosion on the sides of the lithium electrode during continuous charging and discharging. However, for a battery (with a CIHP-SPAN cathode) that is just stored with the same period, no clear corrosion at the lithium side is observed. This corrosion can be assigned to the formation of soluble polysulfides from the cathode side after long-term cycling, which leads to the decay of the long-term cycling performance, especially at a high current density.

**Figure 5:**
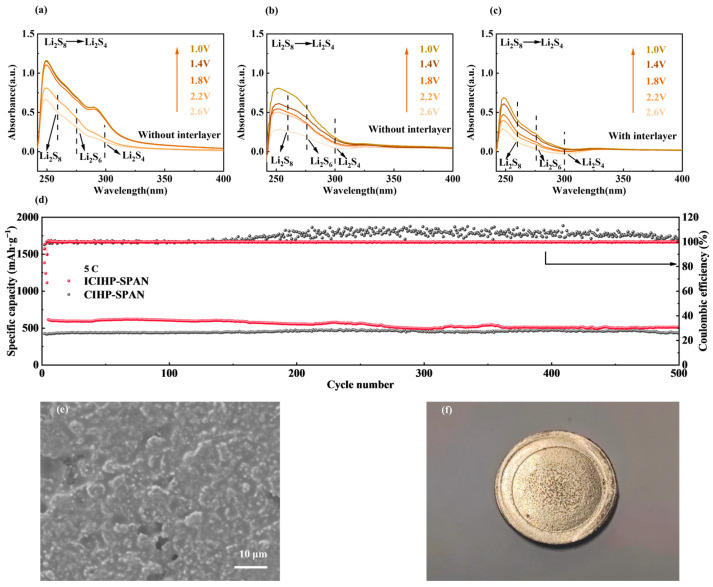
In situ UV-Vis spectrum of the cell with SPAN (**a**) and CIHP-SPAN (**b**) as the cathode. (**c**) In situ UV-Vis spectrum of the cell with CIHP-SPAN cathode and an interlayer of V_2_O_5_/CNFs. (**d**) Long-term cycling performance of CIHP-SPAN and ICIHP-SPAN cathodes at 5 C. (**e**) SEM image and (**f**) photograph of the ICIHP-SPAN cell after 200 cycles.

As shown in the situ UV-vis absorption spectra in Figure 5a–c, during the discharging process, multiple sets of absorption peaks appear in the UV-vis spectra, namely the Li_2_S_8_ absorption peak centered at 260 nm, the Li_2_S_6_ absorption peak centered at 276 nm, and the Li_2_S_4_ absorption peak centered at 300 nm [50]. These peaks indicate the presence of long-chained lithium polysulfides (e.g., Li_2_S_8_ and Li_2_S_6_) in the electrolyte, which can cause a shuttling effect and the corrosion of Li-Metal, as observed in Figure S5 for the CIHP-SPAN samples. The peaks of the CIHP-SPAN samples are reduced from SPAN because of the special hierarchical porous structure that is helpful for the soluble polysulfide inhibition in the cathode side, but a small amount of polysulfide is still observed. To suppress the shuttle effect of soluble polysulfides during electrochemical cycling, a flexible V_2_O_5_ anchored carbon fiber (V-CF) interlayer is applied. The V-CF interlayer was first proposed by us in a lithium-sulfur battery that can provide a quasi-confined cushion space for the stabilization of soluble polysulfides [26]. V_2_O_5_ can physically adsorb the polysulfides on the electrode side and accelerate the electrochemical conversion process, while the carbon fibers help enhance the high current density properties [51,52]. We can see from Figure 5c that the trace of long-chained polysulfides is suppressed after incorporating the V-CF layer, indicating that the V-CF layer effectively inhibits the dissolution of lithium polysulfide [53].

Figure S7a demonstrates the multiplicative cycling performance of CIHP-SPAN, SPAN, V-CF interlayer-modified dense SPAN cells (denoted as ISPAN) and V-CF interlayer-modified CIHP-SPAN cells (denoted as ICIHP-SPAN). Notably, at current densities of 0.5 C, 1 C, and 2 C, the ICIHP-SPAN exhibit a superior capacity property compared to CIHP-SPAN, with the most significant advantage observed at 2 C. Additionally, the ISPAN cell, enhanced by an intermediate layer, outperforms SPAN in terms of multiplier performance. This is attributed to the synergistic effect of the interlayer and the hierarchical porous structure coupled with interpenetrating CNT networks, which leads to a further boost in the cell’s performance. Specifically, the V-CF interlayer immobilizes a small amount of polysulfide on the surface of the cathode. At the same time, the V_2_O_5_ can catalyze the rapid conversion of polysulfides, which improves the reaction kinetics of the battery. At a high cycling current density of 5 C (Figure 5d), the initial specific capacity of the ICIHP-SPAN sample reaches 614.8 mAh g^−1^, which is still as high as 500 mAh g^−1^ after 500 cycles, corresponding to an ultra-low decay rate of only 0.037% per cycle. The V-CF interlayer-modified sample shows a significant enhancement in capacity and leads to a more stable Coulombic efficiency of the cells. High sulfur loading (8.92 mg cm^−2^) cycling (Figure S8) demonstrates a high retained capacity of 674.2 mAh g^−1^ after 50 cycles at 0.1 C for the ICIHP-SPAN cell. Figure 5e,f, show the SEM image and photograph of the ICIHP-SPAN cell after 200 cycles, indicating that the lithium metal surface is much flatter with fewer corrosions than samples without the interlayers. The enhanced redox kinetics and improved cycling stabilities from the current investigations may provide a new route for designing and constructing SPAN-based high-performance batteries that are of great potential for practical applications [54].

## 4. Conclusions

In summary, we reported a flexible CNT-interpenetrating hierarchically porous self-supporting SPAN composite cathode by a simple phase separation process. These cathodes exhibit exceptional electron and ion conductivity due to their unique porous architecture, enabling efficient active material utilization and enhanced cycling performances. Notably, the CIHP-SPAN electrode demonstrates an initial discharge capacity of 1090 mAh g^−1^ at 1 C and maintains as high as 800 mAh g^−1^ after 1000 cycles with a minimal capacity decay rate of 0.026% per cycle. Furthermore, the inclusion of a V-CF interlayer mitigates the shuttling effects from soluble polysulfides during cycling, thus further enhancing the cycle stability. Even at a high current density of 5 C, the obtained cells retain a high specific capacity of 500 mAh g^−1^ after 500 cycles, exhibiting an ultra-low decay rate of 0.037% per cycle. Combined with the comparison of other reported SPAN-based Li-S system cathodes with the present study, the study in this paper still has a great competitive edge (Table S3). This work provides a simple and effective strategy for the synthesis and integration of high-performance SPAN cathode material.

## Data Availability

The data that have been used are confidential.

## Supplementary Materials

The following supporting information can be downloaded at: https://www.mdpi.com/article/10.3390/nano14131155/s1, Figure S1: Optical images of CIHP-SPAN electrode; Figure S2: Bode and Loss tangent plot of SPAN electrode; Figure S3: CV curves of SPAN and CIHP-SPAN composites; Figure S4: Cycling performance comparisons; Figure S5: Morphology characterization and comparison of cycled lithium metal; Figure S6: CV and EIS Comparisons of CIHP-SPAN and ICIHP-SPAN electrode; Figure S7: Rate performances; Figure S8: Cycling performance; Table S1: EA results; Table S2: Comparisons of *R*_ct_ and *R*s; Table S3: comparisons with reported literatures. Refs. [55,56,57,58,59,60,61] are cited in Supplementary Materials.
