# Supplementary material for: Flexible CNT-Interpenetrating Hierarchically Porous Sulfurized Polyacrylonitrile (CIHP-SPAN) Electrodes for High-Rate Lithium-Sulfur (Li-S) Batteries
Source: Nanomaterials (Basel). 2024 Jul 6;14(13):1155. doi: 10.3390/nano14131155 (PMC11242976; doi:10.3390/nano14131155)
Supplement: Supplementary file 1 [file nanomaterials-14-01155-s001.zip › nanomaterials-3074948-supplementary.pdf]

Supporting Information

Flexible CNT-interpenetrating Hierarchically Porous Sulfurized Polyacrylonitrile (CIHP-SAPN) Electrodes for High-Rate Lithium-Sulfur (Li-S) Batteries

Jiashuo Shao [1], Cheng Huang [1], Qi Zhu [1], Nan Sun [1], Junning Zhang [1], Rihui Wang [1], Yunxiang Chen [1], and Zongtao Zhang [1,]*

1 School of Materials Science and Engineering, Zhengzhou University, Kexue Ave 100, Zhengzhou 450001, China; zzusjs2021@163.com (J.S.); hc_1827465@163.com (C.H.); 18143973357@163.com (Q.Z.); sunnan20010315@163.com (N.S.); 13944291225@163.com (J.Z.); wangrihui23@163.com (R.W.); yxchen@zzu.edu.cn (Y.C.)

* Correspondence: ztzhang@zzu.edu.cn.

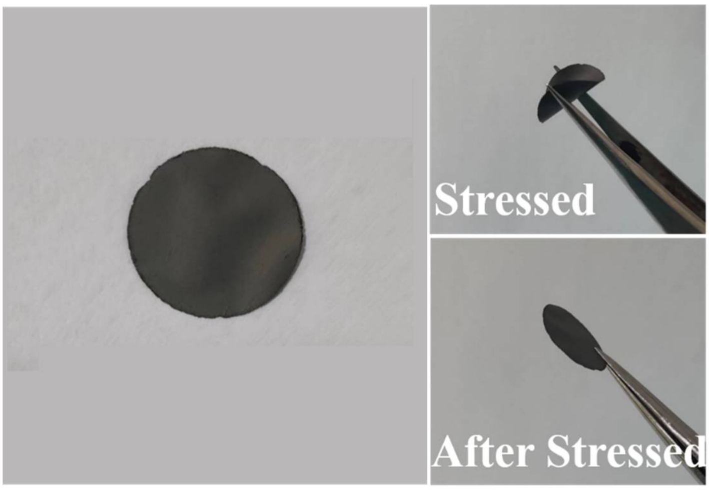

Figure S1. Independent CIHP-SPAN grade sheets and pictures of its flexibility demonstration.

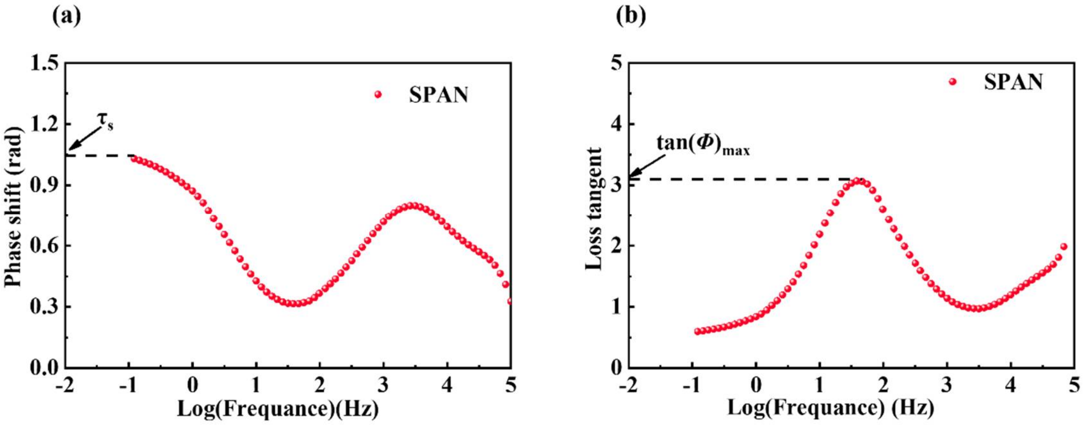

Figure S2. (a) Bode plot of SPAN electrode. (b) Loss tangent plot of SPAN electrode.

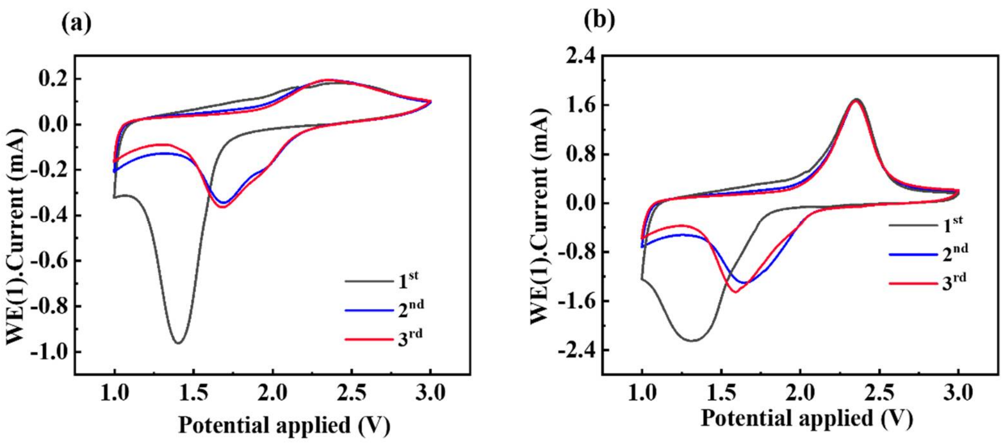

Figure S3. (a) CV curves for the first 3 cycles at a scan rate of 0.1 mV s^{-1} with SPAN and (b) CIHP-SPAN composites.

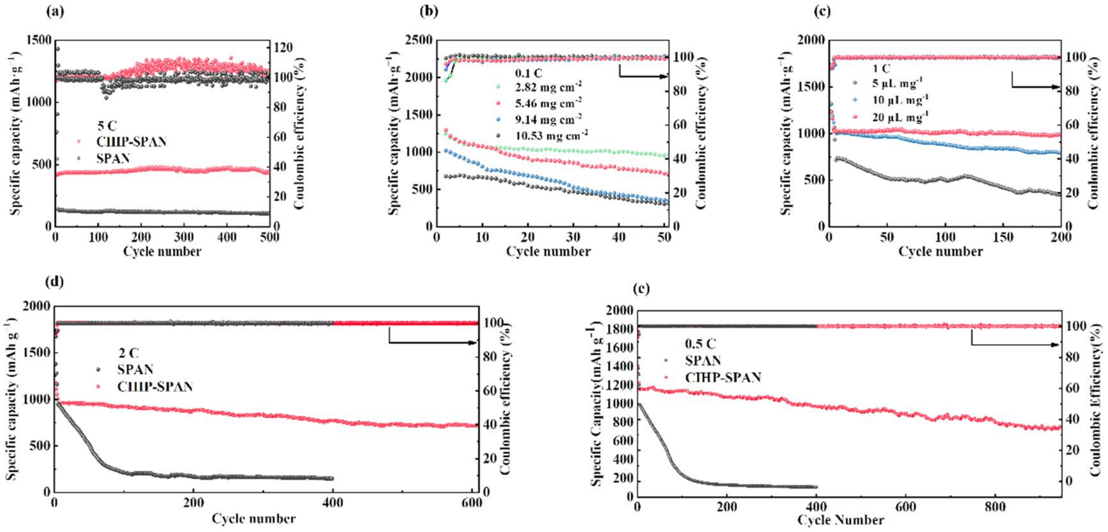

Figure S4. Long cycling performance of SPAN and CIHP-SPAN at (a) 5 C. Cycling performance of CIHP-SAPN at different S loadings (b) and electrolyte contents (c). Long cycling performance of SPAN and CIHP-SPAN at (d) 0.5 C and (e) 2C.

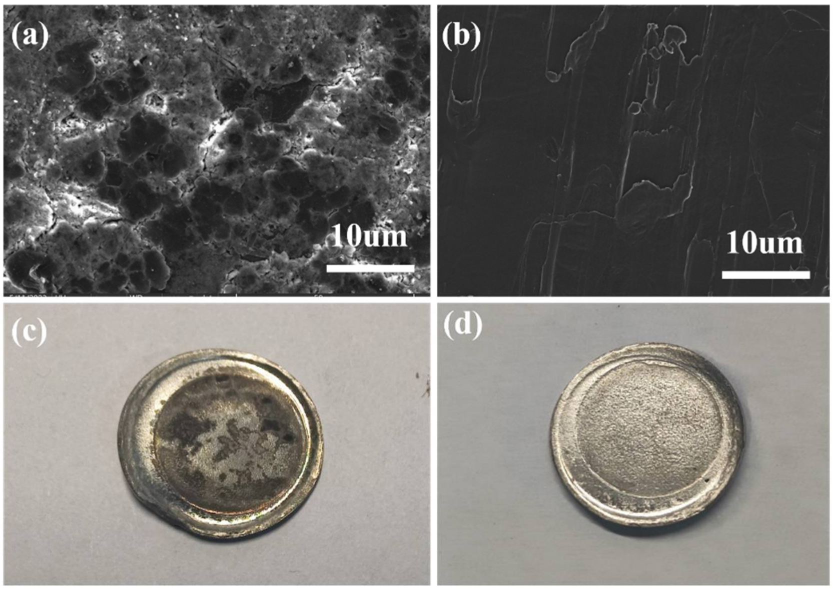

Figure S5. (a) SEM image of the disassembled lithium anode of a coin cell with CIHP-SPAN electrode as the cathode material after 400 cycles at 2C. (b) SEM image of the disassembled lithium anode of a coin cell with CIHP-SPAN as the cathode material after storing for the same time as (a) but without any cyclings. (c) and (d) are the corresponding photographs of (a) and (b), espectively.

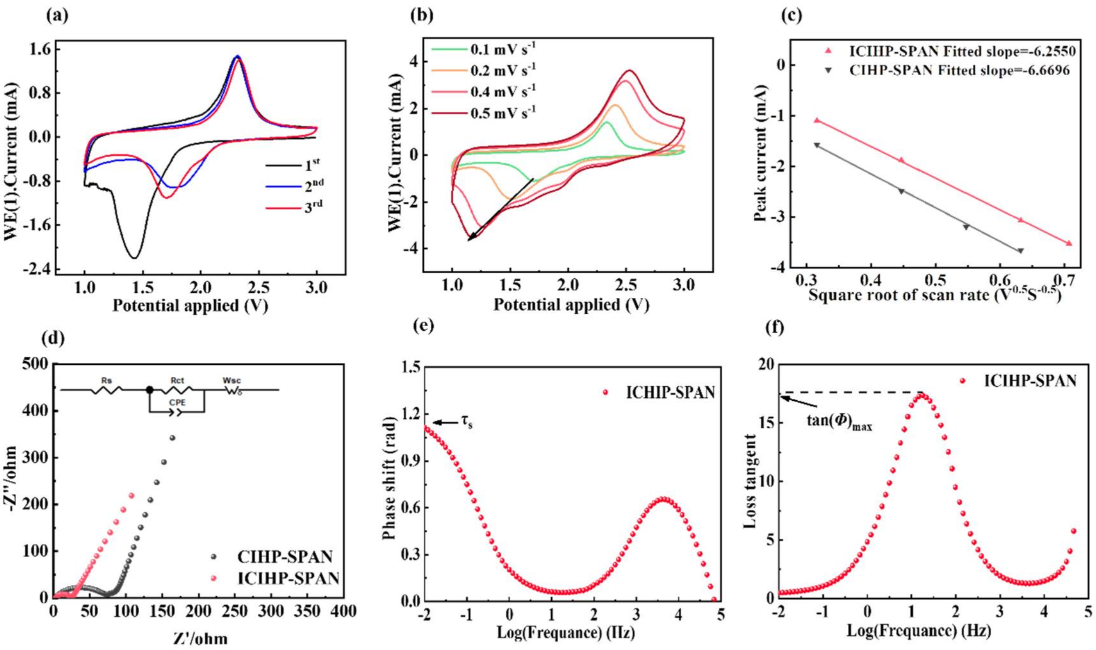

Figure S6. (a) CV curves for the first 3 cycles at 0.1 mV s^{-1} of CIHP-SPAN cathode after added V_2O_5/CNFs interlayer. (b) CV curves of ICIHP-SPAN at different voltage rates and (c) reduction peak-to-peak current versus scan rate. (d) EIS and simulated Nyquist charts for CIHP-SPAN, and ICIHP-SPAN electrodes. (e) Bode plot of SPAN electrode. (f) Loss tangent plot of SPAN electrode.

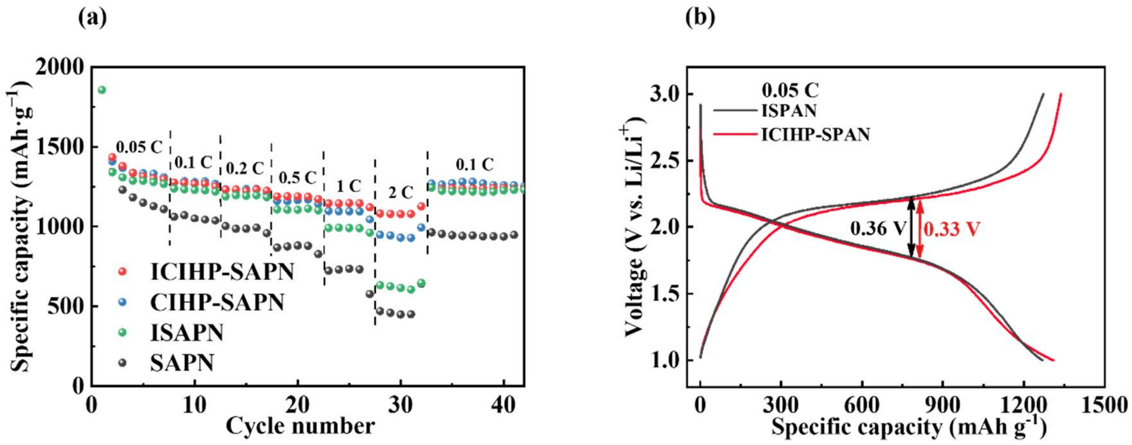

Figure S7. Rate properties (a) of ICIHP-SPAN, CIHP-SAPN ISPAN and SPAN composite cathode materials at specified current densities.(b) The charge and discharge curves of ICIHP-SPAN and CIHP-SAPN at 0.05 C.

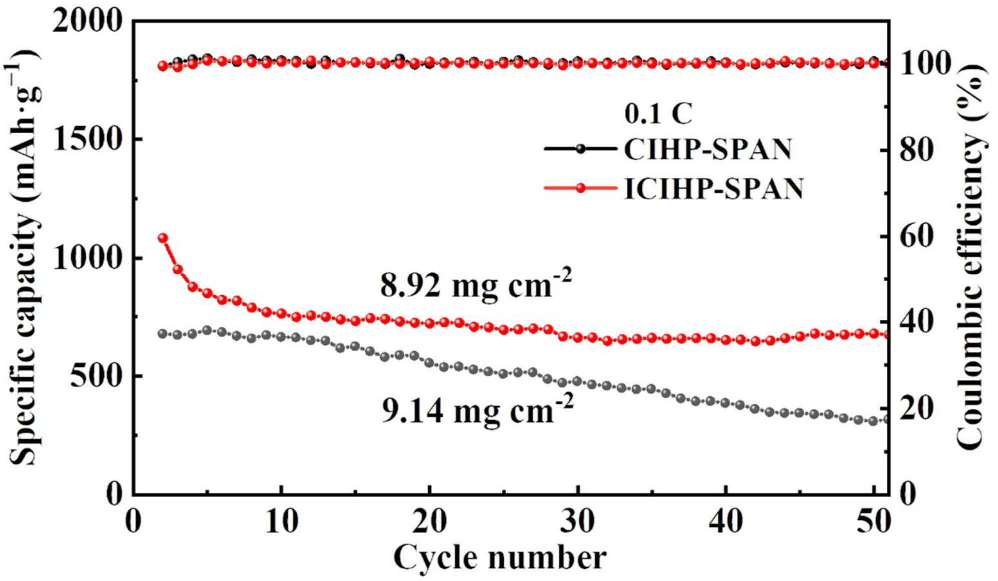

Figure S8. Cycling performance of ICIHP-SAPN at 8.92 mg cm^{-2}

Table S1. EA tests for different types of SPAN and corresponding specific surface areas.

**Types of SPAN**	**Content of sulfur after heat treatment (%)**	**Specific surface area of composites(m^2/g)**
SPAN	45.04	12.8902
CNT-SPAN	38.16	24.6125
CIHP-SPAN	40.28	50.4319

Table S2. (a) Comparison of *Rct* and *Rs* for SPAN and CIHP-SPAN composite electrodes.

**Electrode materials**	***Rs (Ω)***	***Rct (Ω)***
SPAN	2.405	118.4
CIHP-SPAN	2.698	66.45

Table S3. Comparison of other reported SPAN-based cathodes in Li-S systems with this work.

**Electrode materials**	**Current collector**	**S mass loading ($mg\ cm^{-2}$)**	**Current rate**	**Number cycles**	**Fading rate per cycle (%)**	**Refs**
SPANPPy	Freestanding	1.5	1 C	500	0.044	1
$Se_{0.06}$SPAN	Al foil	1 ~ 3	0.4 A g-1	800	0.029	2
SPAN-CNT20	Freestanding	0.9 ~ 1.1	1 C	500	~ 0.032	3
2D-SPAN/G	Al foil	5	0.5 C	500	0.054	4
		10	0.25 C	300	0.07	
CNT_1@SPAN	Freestanding	1	0.5 C	500	0.086	5
$Se_{0.08}S_{0.92}$@pPAN	Al foil	NA	0.4 A g-1	500	0.045	6
SPAN@CDW600	Freestanding	2.1	0.1 C	500	0.03	7
3DHG/PS	Freestanding	6.11	2 C	1500	0.012	8
SPAN/CNT-500	Freestanding	NA	0.4 A g-1	200	0.02	9

SPAN/RGO	Al foil	NA	0.1 C	200	~ 0.1	[10]
S/DPAN/MWCNT	Freestanding	3	0.2 C	260	0.053	[11]
Porous PAN/S	Freestanding	-	2C	500	-	[12]
Cihp-SPAN	Freestanding	~1.5	1 C	1000	0.026	
ICIHP-SPAN	Freestanding	~1.5	5 C	500	0.037	This work
ICIHP-SPAN	Freestanding	~1.5	2 C	610	0.022	

Reference

1. Yi, Y.; Hai, F.; Guo, J.; Gao, X.; Chen, W.; Tian, X.; Tang, W.; Hua, W.; Li, M., Electrochemical Enhancement of Lithium-Ion Diffusion in Polypyrrole-Modified Sulfurized Polyacrylonitrile Nanotubes for Solid-to-Solid Free-Standing Lithium–Sulfur Cathodes. *Small* **2023**.
2. Xin; Chen; Linfeng; Peng; Lihui; Wang; Jiaqiang; Yang; Zhangxiang; Hao, Ether-compatible sulfurized polyacrylonitrile cathode with excellent performance enabled by fast kinetics via selenium doping. *Nature Communications* **2019**.
3. Razzaq, A. A.; Yao, Y.; Shah, R.; Qi, P.; Miao, L.; Chen, M.; Zhao, X.; Peng, Y.; Deng, Z., High-Performance Lithium Sulfur Batteries Enabled by a Synergy between Sulfur and Carbon Nanotubes. *Energy Storage Materials* **2018**, 194-202.
4. Kim, H.; Hwang, J. Y.; Bang, S.; Jung, H. G.; Sun, Y. K., Geometrical engineering of a SPAN–graphene composite cathode for practical Li–S batteries. *Journal of Materials Chemistry A*.
5. Hu, X.; Jiang, H.; Hou, Q.; Yu, M.; Jiang, X.; He, G.; Li, X., Scalable SPAN Membrane Cathode with High Conductivity and Hierarchically Porous Framework for Enhanced Ion Transfer and Cycling Stability in Li-S Batteries.
6. Wang, L.; Chen, X.; Li, S.; Yang, J.; Sun, Y.; Peng, L.; Shan, B.; Xie, J., Effect of eutectic accelerator in selenium-doped sulfurized polyacrylonitrile for high performance room temperature sodium-sulfur batteries. *Journal of Materials Chemistry A* **2019**.
7. Sabet, S. M.; Sapkota, N.; Chiluwal, S.; Zheng, T.; Clemons, C. M.; Rao, A. M.; Pilla, S., Sulfurized Polyacrylonitrile Impregnated Delignified Wood-Based 3D Carbon Framework for High-Performance Lithium–Sulfur Batteries. **2023**.
8. Wang, T.; Zhang, Q.; Zhong, J.; Chen, M.; Lu, B., 3D Holey Graphene/Polyacrylonitrile Sulfur Composite Architecture for High Loading Lithium Sulfur Batteries. *Advanced Energy Materials* **2021**.
9. A, H. L.; A, W. X.; A, W. X.; A, L. W.; B, T. L. A., Controllable synthesis of sulfurized polyacrylonitrile nanofibers for high performance lithium–sulfur batteries. *Composites Communications* **2021**.
10. Li, J.; Li, K.; Li, M.; Gosselink, D.; Zhang, Y.; Chen, P., A sulfur–polyacrylonitrile/graphene composite cathode for lithium batteries with excellent cyclability. *Journal of Power Sources* **2014,** *252* (apr.15), 107-112.
11. Mentbayeva, A.; Belgibayeva, A.; Umirov, N.; Zhang, Y.; Taniguchi, I.; Kurmanbayeva, I.; Bakenov, Z., High performance freestanding composite cathode for lithium-sulfur batteries. *Electrochimica Acta* **2016,** *217*, 242-248.
12. Wang, K.; Ju, S.; Gao, Q.; Xia, G.; Yu, X., Porous sulfurized poly(acrylonitrile) nanofiber as a long-life and high-capacity cathode for lithium–sulfur batteries. *Journal of Alloys and Compounds* **2020**, 158445.
